# Possibility of diagnosing rotator cuff tears in areas with scarce medical resources: can non-standard anteroposterior radiographs accurately predict rotator cuff tears?

**DOI:** 10.3389/fmed.2024.1484851

**Published:** 2024-10-15

**Authors:** Feng Xiong, Wenbin Zhang, Feilong Lu, Jie Feng, Lu Wang, Yulu Xiang, Yongtao Wang, Yimei Hu

**Affiliations:** ^1^Department of Orthopedics, Jiang’an County Traditional Chinese Medicine Hospital, Yibin, Sichuan, China; ^2^School of Clinical Medicine, Chengdu University of Traditional Chinese Medicine, Chengdu, Sichuan, China; ^3^College of Medicine, Southwest Jiaotong University, Chengdu, Sichuan, China; ^4^Department of Rehabilitation, Affiliated Hospital of Inner Mongolia Medical University, Hohhot, China

**Keywords:** radiology, rotator cuff tear, diagnosis, nonstandard anteroposterior radiographs, medical resources

## Abstract

**Background:**

Due to the scarcity and high cost of MRI in resource-constrained regions, prompt diagnosis and treatment of rotator cuff tears remain problematic in these areas. Therefore, extensive research has been conducted to predict rotator cuff tears using simple and affordable anteroposterior radiographs. It remains unclear whether non-standard anteroposterior radiographs would have a notable impact on the preciseness of the diagnosis.

**Method:**

We analyzed patients treated for shoulder pain at hospitals. These patients underwent shoulder joint MRI and standard anteroposterior radiographs, were categorized into those with rotator cuff tears and a control group. We assessed whether the radiographs were standard anteroposterior radiographs using classification criteria from previous studies. Three assessors independently measured the acromiohumeral interval, upwards migration index, acromion index, critical shoulder angle, and double-circle radius ratio in radiographic images. The intraclass correlation coefficient and receiver operating characteristic curves were used to assess measurement reliability and predictive capabilities of each predictive method for rotator cuff tears.

**Results:**

This study included 102 non-standard radiographs that met the research criteria for the measurement and analysis. The intragroup correlation coefficients for the acromiohumeral interval, upwards migration index, and double-circle radius ratio were above 0.7 (0.77, 0.71, 0.76), while those for the acromion index and critical shoulder angle exceeded 0.8 (0.86 and 0.87). In non-standard radiographs, the double-circle radius ratio reliably predicted rotator cuff tears (*p* < 0.05), contrary to the other methods (*p* > 0.05). The areas under the receiver operating characteristic curves of the double-circle radius ratio, estimated by the three researchers for rotator cuff tears.

**Conclusion:**

This study found that non-standard radiographs significantly impaired the diagnostic performance of the acromiohumeral interval, upwards migration index, acromion index, and critical shoulder angle. Only the double-circle radius ratio maintained its predictive power (although this diminished capability may fall short of clinical relevance) and demonstrated high applicability. These findings indicate the need for researchers to prioritize the quality of radiographs and focus on reducing the sensitivity of the prediction method in relation to radiograph quality. The capability exhibited by the double-circle radius ratio warrants further investigation, to facilitate a simplified diagnosis of rotator cuff tears.

## Introduction

The incidence of rotator cuff tears (RCTs) is associated with age. Approximately 25 and 50% of individuals between 60 and 80 are affected by full-thickness RCTs (1). The number of patients with RCTs gradually increases with the global aging population. An Italian study estimated that healthcare costs for rotator cuff surgery will exceed one billion euros by 2025 (2). RCTs are imposing an increasingly heavy economic burden (3). Although appropriate treatments can be adopted for different stages of the disease, even for massive irreparable rotator cuff tears, reverse shoulder arthroplasty can still be chosen as a treatment. However, compared with early interventions, it faces a higher risk of complications, revisions, and infections (4–6). Prompt intervention for RCTs is beneficial based on therapeutic efficacy and cost-effectiveness. MRI with high diagnostic accuracy is essential for early diagnosis to achieve this objective (7); however, it is relatively expensive. Analysis of insurance data revealed that the cost of magnetic resonance imaging (MRI) examination accounted for a significant amount of preoperative costs (8).

In regions lacking medical resources, access to MRI is limited and frequently unaffordable. Consequently, afflicted individuals often do not receive prompt and appropriate treatments, resulting in disease progression, reduced quality of life, work capacity, and increased societal medical expenses. Therefore, several studies aimed to utilize simpler radiographs for preliminary RCT screenings, consistent with the resources available in primary healthcare settings (9). Numerous diagnostic methods have been proposed to identify characteristic changes in shoulder joint diseases (including RCTs) from scapula anteroposterior (AP) radiographs. Notable instances include the acromiohumeral interval (AHI), upward migration index (UMI), acromion index (AI), critical shoulder angle (CSA), and double-circle radius ratio (DRR) (10–18). These predictive methods are closely related to RCTs. These indicators are used to predict RCT occurrence, inform treatment decisions, and evaluate the therapeutic effect after rotator cuff tear surgery (19–22).

The inherent bidimensional imaging property of conventional radiographic films often leads to a superimposed fusion of tridimensional anatomical structures in the images. Measurements obtained from these radiographs can be significantly affected by the projection angle and quality of the radiographs (23, 24). Standard AP radiographs of the scapula could theoretically minimize such overlap and blurred boundaries, thereby enhancing data accuracy. Nevertheless, non-standard AP radiographs are unavoidable in clinical practice, potentially leading to measurement errors. Numerous existing predictive methods, including AHI, UMI and DRR, have not demonstrated their efficacies when utilized on non-standard AP radiographs.

Consequently, investigating whether these methods maintain their predictive power under these circumstances is imperative. Assessing the accuracy of these predictive methods on non-standard AP radiographs and investigating methods less affected by radiograph quality may enhance the clinical applications of radiographs in predicting RCTs. This may facilitate more diagnostic and treatment options for RCTs in areas with limited medical resources.

## Methods

In this study, medical records were compiled from patients who underwent AP radiographs and MRI scans on the same side shoulder with a gap no longer than 2 months. These records, collected from January 2017 to April 2023, were obtained from Sichuan Province Orthopedic Hospital and Sichuan Province Traditional Chinese Medicine Hospital. Diagnoses were made using a 3.0 T MRI, the gold standard for identification (25, 26).

Three researchers utilized Digimizer software to measure the AHI, UMI, AI, CSA, and DRR data in the collected AP radiographs. Through these objective indicators, the forecast capability of the aforementioned methods was assessed in non-standard AP radiographs. This study adhered to ethical standards set by the Medical Ethics Committee of the Affiliated Hospital of Chengdu University of Traditional Chinese Medicine, and subsequently received ethical approval (Ethics approval number 2024KL-026).

### Research subject

The participants of this research were patients who reported shoulder discomfort at the Sichuan Province Orthopedic Hospital and Sichuan Province Traditional Chinese Medicine Hospital. These patients were sorted into two categories based on their MRI diagnoses: the RCTs group and a control group, the latter without RCTs. The former was subdivided into groups with partial and full-thickness RCTs, with the latter group including cases of rotator cuff rupture. The specific inclusion and exclusion criteria are enumerated below.

#### Inclusion criteria

(1) AP radiographs of the shoulder joint; (2) MRI scans of the same shoulder joint, conducted for diagnostic purposes; (3) Patient’s age is 18 years or more; and (4) Radiologists’ confirmation regarding the clarity and completeness of the imaging data.

#### Exclusion criteria

(1) A time gap exceeding 2 months between the AP radiograph and MRI scan; (2) Any prior shoulder surgery; (3) A previous account of shoulder fracture, tumor, or habitual shoulder dislocation; (4) Conditions such as severe shoulder arthritis, calcifying tendinitis, or displacement or rupture of the long head of the biceps brachii; (5) Moderate to severe damage to the humeral head, the glenoid cavity, or acromion structure; (6) Limited active and passive shoulder movement, with high suspicions of Periarthritis; and (7) Trauma-induced shoulder discomfort.

### Imaging evaluation

The MRI scans were all conducted using a 3.0 T MRI system (GE Discovery MR750 3.0 T). Patients were positioned supine with their arms neutrally placed by their sides and palms facing upwards. For the AP radiographs (Carestream DR: EVOLUTION VX3733-SYS), patients stood with their arms neutrally positioned and hands in anatomical posture. Their shoulder was directed 35–45° towards the X-ray plate, ensuring the plane of the scapula paralleled the dark box. The patient was stationed approximately 120 millimeters away from the film and the central X-ray beam was angled towards the head-to-tail line at a 15–20° inclination.

**Figure 1:**
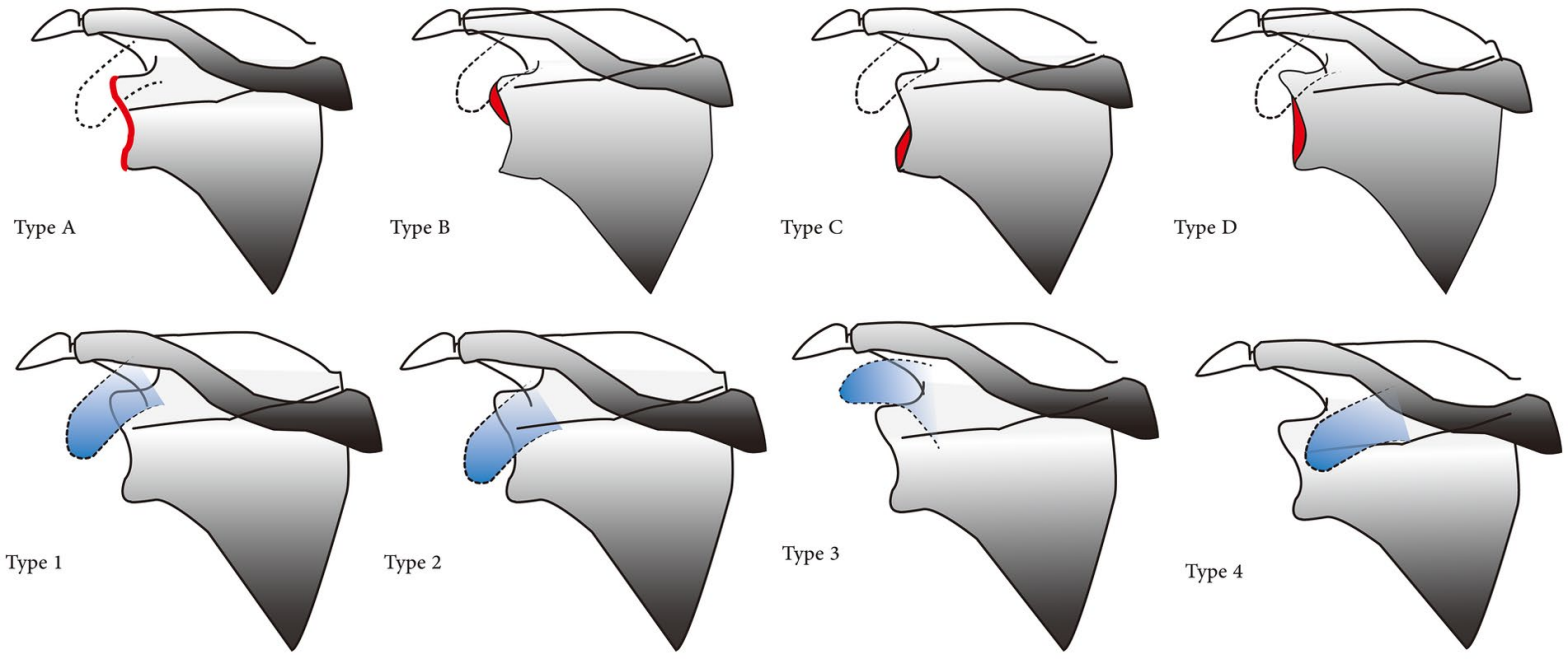
Classification criteria for AP radiographs. The anatomy of the glenoid rim is classified into four (A–D) and overlaps the position of the upper glenoid rim and coracoid process in four types (1–4). Type A: Overlapping without a discernible double contour shape; Type B: Exhibits a droplet-shaped double contour on the upper rim; however, this accounts for<50% of height of the glenoid rim; Type C: The double contour position on the lower glenoid rim and constitutes <50% of the height of the glenoid rim; Type D: Double contour that >50% of the glenoid rim. Type 1: The coracoid process overlaps with the upper glenoid rim, or the lower edge of the coracoid process aligns with the glenoid rim. Type 2: Absence of overlap between the upper glenoid rim and the coracoid process, with the coracoid process positioned below the upper glenoid rim or the upper edge. Type 3: No overlap was observed between the upper glenoid rim and the coracoid process, with the coracoid process situated above the glenoid rim. Type 4: The coracoid process is located on the inner side of the glenoid rim, and the edge of the coracoid process does not extend beyond the glenoid rim.

**Figure 2:**
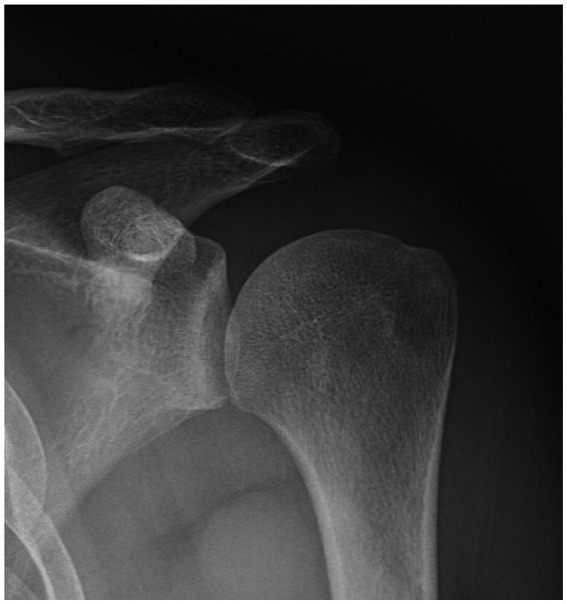
The coracoid process located on the inner side of the glenoid rim,and the edge of the coracoid process does not extend beyond the glenoid rim.

All imaging data were diagnosed by experienced radiologists through a combination of MRI, examination request forms, and outpatient medical records. Eligible patients, in accordance with the defined inclusion and exclusion criteria, were included for the study. Information such as age, sex, imaging date, and diagnostic outcomes were collected. AP radiographs were subsequently classified. The study employed the Suter-Henninger categorization scheme for scapulas, where Type A1 or C1 radiographs were identified as standard AP radiographs (Figure 1) (23, 24). All other categories were deemed non-standard. This study additionally discovered a previously unclassified variation, Type 4, which was characterized by a complete overlay of the coracoid process in the imaging of the scapular neck (Figure 2).

### Data collection

**Figure 3:**
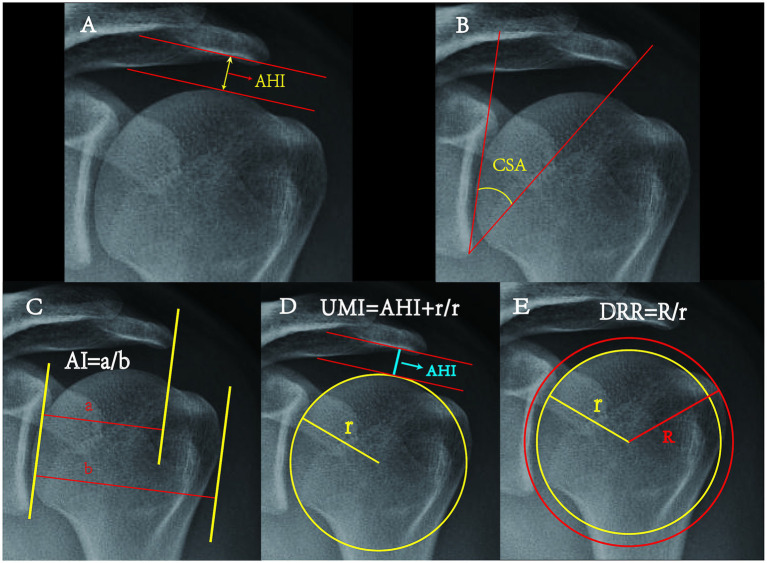
Schematic diagram of each measurement method. AHI: The minimum distance from the subcortical surface of the acromion to the humeral head (33). CSA: The angle formed by the line connecting the upper and lower rims of the glenoid, and the line extending from the lower glenoid to the outside edge of the acromion (34). AI: The ratio of two distances- the rim of the glenoid to the acromion (a) and from the rim of the glenoid to the outer edge of the humeral head (b) (24). UMI: The division of the distance from the geometric center of the humeral head to the subcortical surface of the acromion by the radius of the humeral head (33). DRR: The ratio between the outer circle radius R and the inner circle radius r, where the inner circle is the optimal fit for the humeral head, and the outer circle is a concentric circle intersecting the greater tubercle of the humerus (14).

Three orthopedic doctors, boasting substantial diagnostic acumen and clinical experience, independently evaluated the AHI, UMI, AI, CSA, and DRR through the use of Digimizer software subsequent to rigorous training. All data, including AHI measurements taken in millimeters, were recorded to two decimal places to enhance precision. The magnification range for the image measurement was set between 500 and 2,000% to further advance accuracy (Figure 3).

### Statistical analysis

Data collected for this retrospective study was processed using SPSS 26.0 software, summarized as mean ± standard deviation (SD) with *α* = 0.05 as the test level. Any *p*-value less than 0.05 reflected statistical significance. The chi-square test was employed to scrutinize the gender difference across each group. Age and measurement data were evaluated using *t*-test and one-way analysis of variance (ANOVA) when they followed a normal distribution, and a rank-sum test was used otherwise. The intra-group correlation coefficient (ICC) was used to analyze the congruence of measurements across different researchers where values less than 0.2, between 0.2 and 0.4, between 0.4 and 0.6, within the range of 0.6 to 0.8, and between 0.8 to 1.0 signified poor, general, medium, strong and very strong consistency, respectively. The correlation between measured values and RCTs was evaluated using Eta-squared (η^2^) in ANOVA. The capability of various predictive methods was assessed by the area under curve (AUC) of receiver operating characteristic (ROC).

## Results

### Basic information

This study included 107 samples that met the inclusion criteria, including 102 non-standard AP and 5 standard radiographs. In the non-standard AP radiographs, one was classified as type B, while the remainder were categorized as type D, with type D1 constituting the vast majority. The non-standard AP radiographs were measured. The results revealed no significant difference in the sex ratio among the various groups. The mean age of the participants in the control group (34.09 ± 11.89 years) was significantly lower than that of those in the RCTs group (52.07 ± 11.51 years) (Table 1).

**Table 1:** Basic information.

	Control	RCT	Subgroup	
			Partial	Full-thickness
Number	43	59	46	13
Male/Female	17/26	25/34	17/29	8/5
Age(year)	34.09 ± 11.89	52.07 ± 11.51^a^	50.96 ± 11.88^a^	56 ± 9.44^a^

a A significant difference when compared with the control group, with *p* < 0.05.

**Figure 4:**
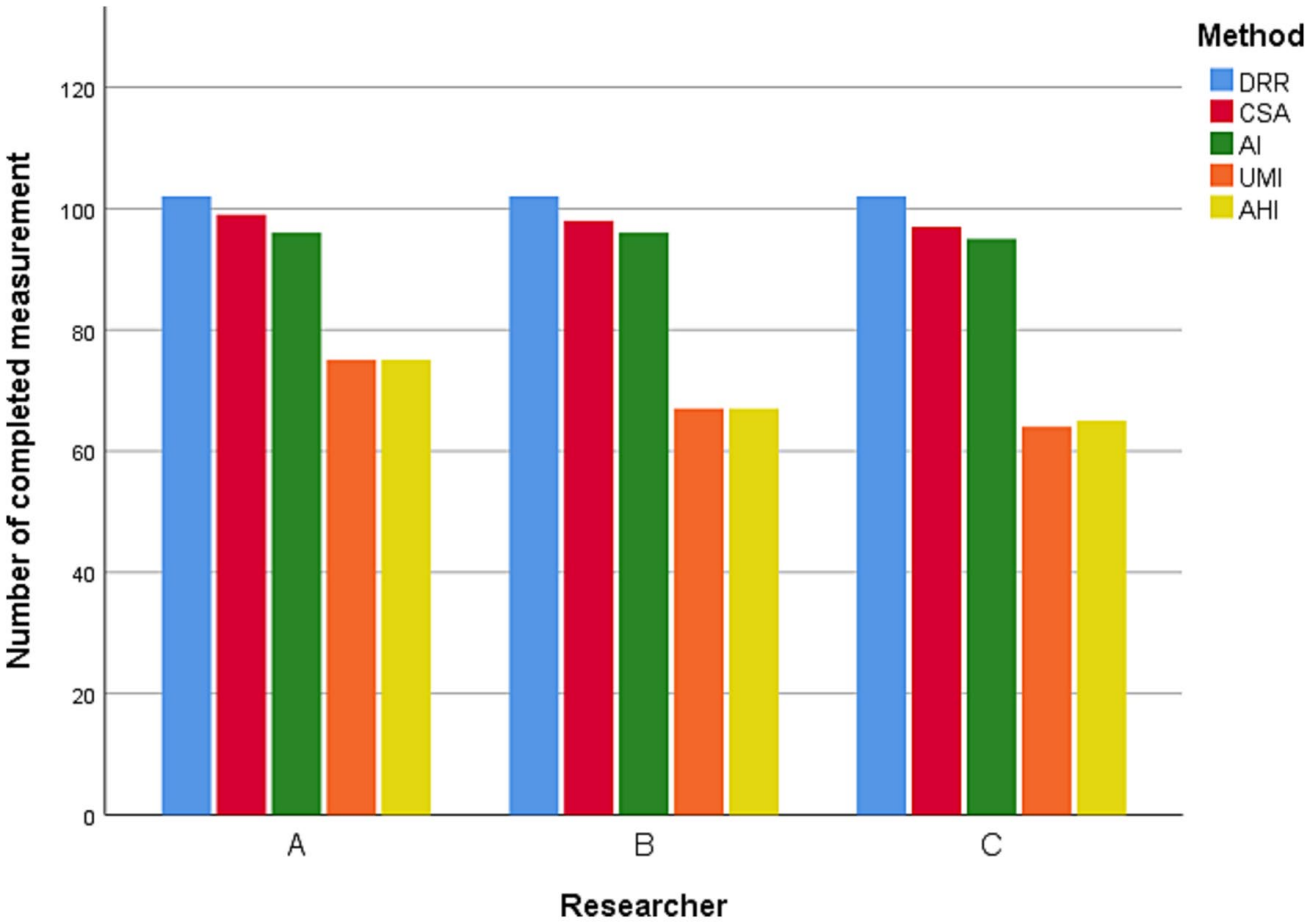
Total number of completed measurements.

Bone boundary evaluation was difficult due to non-standard projection angles and anatomical variations. Accurate measurement of radiographs across all prediction methods was unfeasible. DRR successfully measures all radiographs of the five prediction methods, while CSA and AI assess the majority. Many radiographs did not meet the criteria for AHI and UMI method (Figure 4). ICC analysis revealed a significant level of consistency among the data measured by three researchers (Table 2).

**Table 2:** Consistency of data among observers—ICC.

AHI	DRR	UMI	CSA	AI
0.774	0.764	0.713	0.874	0.855

### Ability to predict RCTs based on non-standard X-ray films

Statistical analysis revealed significant differences between the control and RCT groups along and their sub-groups as detected using DRR (*p* < 0.05). However, no significant differences were observed in other prediction methods (*p* > 0.05) (Table 3). Further analysis revealed that the eta-squared values from the two researchers demonstrated a moderate correlation between RCTs and DRR. Additionally, the data from the other researcher revealed a moderate correlation solely between full-thickness RCTs and DRR (Table 4).

**Table 3:** Measurement data.

Method	Assessors	Control	RCT	Subgroup	
				Partial	Full-thickness
AHI	A	8.44/2.22	8.37/1.45	8.40/1.28	8.25/1.94
	B	8.92/2.21	8.51/1.61	8.62/1.33	8.10/2.42
	C	9.28/1.81	8.50/1.63	8.55/1.39	8.33/2.34
DRR	A	1.16/0.06^b^	1.21/0.06^a^	1.20/0.06	1.21/0.05
	B	1.15/0.08^b^	1.20/0.07^a^	1.20/0.05	1.23/0.07
	C	1.16/0.05^b^	1.20/0.06^a^	1.19/0.06	1.22/0.06
UMI	A	1.35/0.10	1.36/0.06	1.36/0.05	1.35/0.08
	B	1.38/0.03	1.36/0.06	1.37/0.05	1.34/0.03
	C	1.39/0.10	1.36/0.06	1.36/0.05	1.36/0.09
CSA	A	38.18/5.59	38.25/4.27	38.00/4.37	39.17/3.93
	B	36.37/5.29	36.46/4.06	36.37/4.29	36.80/3.27
	C	36.29/5.18	36.31/3.90	36.29/4.11	36.36/3.24
AI	A	0.74/0.09	0.74/0.08	0.74/0.08	0.75/0.08
	B	0.73/0.09	0.73/0.08	0.73/0.08	0.73/0.09
	C	0.73/0.08	0.73/0.08	0.73/0.08	0.75/0.10

a A significant difference between the control group and the RCTs group, *p* < 0.05.

b A significant difference between the control group and each subgroup, *p* < 0.05.

**Table 4:** Correlation between DRR and rotator cuff tear (*η^2^*).

Assessors	RCT	Partial	Full-thickness
A	0.135	0.125	0.141
B	0.140	0.116	0.160
C	0.011	0.014	0.155

AUC analysis revealed that four of the five prediction methods (AHI, UMI, CSA, and AI) used by the three researchers lack diagnostic capability (*p* > 0.05) for non-standard AP radiographs. The DRR method demonstrated a certain level of diagnostic capability, with an AUC for RCTs exceeding 0.6; the full-thickness RCTs prediction achieved an AUC above 0.7 (Table 5).

**Table 5:** AUC of DRR.

ROC curve	RCT			Subgroup					
				Partial			Full-thickness		
Assessors	A	B	C	A	B	C	A	B	C
AUC	0.71	0.70	0.66	0.69	0.68	0.64	0.77	0.77	0.73
*p*	0.00	0.00	0.01	0.00	0.00	0.02	0.00	0.00	0.01

## Discussion

This study found no significant differences in AHI, UMI, CSA, and AI between the RCTs and control groups based on non-standard AP scapula radiographs. This indicated that non-standard AP radiographs significantly affect the assessment of RCTs. Despite this, DRR demonstrated a certain level of diagnostic capability, good measurement consistency, and outstanding applicability, and it highlighted the unique benefits of the DRR measurement method.

### Quality of radiographs affects predictive accuracy

Easy and accurate screening methods could enhance the capacity of primary healthcare institutions to perform preliminary diagnosis and treatment of RCTs. This is particularly beneficial for the early identification of diseases in resource-deprived regions, resulting in prompt interventions and enhanced treatment outcomes. Additionally, this could mitigate the need for expensive MRIs, thereby reducing diagnostic costs. Consequently, the diagnosis of RCTs using radiographs has been investigated. Despite using radiographs of varying quality in clinical settings, previous studies have overlooked the impact of radiograph quality on diagnostic efficacy. Consequently, it is essential to evaluate the effect generated by radiograph quality.

Previous studies have reported a solid association between the prediction methods employed in this study and RCTs. In a previous study that involved 75 MRI-confirmed RCT samples, UMI values below 1.38 and 1.3 were significant contributors to tear size (11). AHD < 6 mm often indicates RCTs (10). A CSA value above the average in the Turkish population is significantly associated with an increased incidence of degenerative, full-thickness RCTs (27). A preliminary investigation of a new measurement technique reported that a DRR value >1.38 increased the likelihood of RCT occurrence by eleven-fold (14).

This study found that non-standard AP scapular radiographs significantly impaired RCT prediction accuracy. Despite evidence of strong correlations between the employed predictive methods, and RCTs, no significant differences were identified in AHI, UMI, CSA, and AI between RCT and control groups, indicating the inability of these indicators to predict RCTs. This significantly contrasts with previous studies using standard AP radiographs, indicating that non-standard radiographs significantly affect the predictive capacity of AHI, UMI, CSA, and AI.

These findings are consistent with those of previous research conducted by Yiyong Tang and Thomas Suter’s team. Suter et al. discovered that minor variations in projection angle resulted in changes to the CSA. Even minimal deviations of approximately 5° in anteversion produced a CSA greater than 2° from accurate AP view (23). Tang’s study discovered a significant reduction in AUC in CSA and AI between RCTs and control groups due to non-standard radiographs, despite the mean CSA value not exhibiting a significant difference between the two groups (*p* = 0.536) (24). Herein, the non-standard radiographs affected the predictive ability of RCTs, rendering AHI, UMI, CSA, and AI unable to retain their predictive capability.

Despite this, the data obtained from non-standard radiographs exhibited reasonable consistency among different assessors, particularly in CSA and AI. This may be attributed to our team exclusively assessing radiographs that displayed the corresponding anatomical structure. The methodology diminished the measurement error.

### DRR: commendable capability to withstand disturbances affecting the quality of radiographs

We observed significant discrepancies in DRR. It maintained the potential to predict RCTs in non-standard radiographs. Significant deviations were observed in the measured values of the RCTs group and its subsets compared to the control group. The total values of the RCTs group were higher than those of the control group, indicating a potential association between larger humerus nodules and RCTs (28). Previous retrospective studies reported an exceptional predictive capacity of DRR: The AUC was >0.8 in a standard radiograph, with sensitivity and specificity measured at >0.70 and 0.80, respectively (14, 15). In an extensive meta-analysis focused on MRI diagnosis of RCTs, the sensitivity and specificity of MRI for RCTs detection were 0.84 and 0.86, respectively (26). Preliminary studies on DRR reported that its diagnostic performance in standard radiographs is close similar to that of MRI.

This research demonstrated that the AUC of RCTs using DRR was >0.6, based on non-standard radiographs, demonstrating a certain level of diagnostic capability. However, the AUC value of DRR in this study was relatively small, with considerable possibility of errors, and it could not prove its accurate diagnosis for RCTs, lacking clinical significance (29). Still, reasonable inferences can be made through these changes: compared to the models in previous studies, the AUC has significantly decreased. Compared with other prediction methods, the damage DRR suffers in non-standard AP radiographs is less significant.

This study identified the accurate delineation of the inferior margin of the acromion as challenging due to anatomical variation and inferior radiograph quality, thereby rendering AHI and UMI ineffective in several samples. However, CSA and AI exhibited better measurability, with DRR measurable in all samples. Consequently, DRR has a higher chance of being used in most AP radiographs.

Among non-standard AP radiographs, DRR was the only indicator that maintained a certain diagnostic capability, demonstrating better usability than other indicators. This difference may be due to DRR measurement excluding two anatomical features prone to errors (the anatomy of the shoulder peak and glenoid cavity). The change of DRR does not depend on the relationship between the shoulder peak and glenoid cavity but solely on the impact of the humeral head and the major humeral tubercle, which reduces the interference. In contrast to the shoulder peak and glenoid cavity, ensuring the proper imaging of the major humeral tubercle is easier. Simultaneously, the form of the ratio further reduces the impact of absolute value change. However, the effect of imaging angle or upper limb position changes on DRR values has not been extensively investigated.

In conclusion, utilizing non-standard radiographs as the basis, the diagnostic ability of DRR, data consistency, and extensive applicability make it a potentially more efficient prediction method. DRR has a good predictive ability and requires less image quality, which reduces its application difficulty in clinical work and fits the medical conditions in technology-underdeveloped and resource-limited regions. This research is the first to employ radiographs for DRR measurements. Previous studies utilized three-dimensional models developed using computed tomography scans, reported the ability of DRR to resist the negative impacts of radiograph quality, suggested its potential advantage in real-world clinical environments. The potential advantages highlight the feasibility of the DRR approach as a predictive tool, necessitating further investigation, validation, enhancement, and utilization in RCT diagnosis.

### How can measurement errors caused by image quality be reduced?

Standard true AP radiographs primarily depend on the association between the coronoid, shoulder peak, and glenoid. The standard projection prevents deviations in the true relative position between the shoulder joint structures. Additionally, the values of that method will vary significantly based on the body position and posture of the patient and projection direction. The measurement accuracy depends on the spatial relationship between the scapula and the radiographic beam. Comparing ultrasonography evaluation with the true AP standard, primarily in shoulder AP, revealed that AHI exhibited the best accuracy in the standard true AP position (30). Standing or supine affects the measured values; the AHI of the supine radiograph was significantly lower than the upright shoulder radiograph, and the average difference between the AHI observed in the supine radiograph and MRI is significantly lower than the upright (31). This suggests that although these methods are cheaper and more convenient, there are specific application conditions, and research and application need to be performed under standard projections and unified body positions.

Minimizing the discrepancy between measured outcomes and actual characteristics is crucial for clarifying the reliability of radiographs in predicting RCTs. Herein, the advantage of DRR was its reduced susceptibility to the quality of radiographic images. The research team believed that the fundamental aspect was to minimize the requisite components of the measurement method and select anatomic landmarks with less variation. Another concept was to optimize projection methods to improve the quality of radiographs. it is necessary to improve radiologists’ understanding of radiograph quality and their professional level. Tridimensional imaging technology can provide freedom of measurement angle and prevent projection difficulty. Using machine learning to quickly assess the quality of each image and eliminate unqualified radiographs can prevent inappropriate application of predictive methods.

### Limitations

This study has several limitations. First, the insufficient research in this area requires extensive research and a larger sample size to elevate evidence quality. Furthermore, it is necessary to refine the categorization based on the location and extent of the RCTs further to identify more specific variations. Second, the diagnostic modality. Despite the high accuracy of 3.0 T MRI in identifying RCTs (25, 32), future studies should integrate arthroscopic examination or MR Arthrography to support the diagnosis and enhance diagnostic precision.

## Conclusion

This study demonstrated that the quality of radiographs significantly affects AHI, UMI, AI, and CSA predictive ability for RCTs, indicating the crucial need for researchers to utilize high-quality radiographs in their studies. We must focus on the sensitivity of predictive methods against variations in radiograph quality. Only the DRR demonstrated a certain level of diagnostic capability under non-standard radiograph conditions, indicating superior adaptability. This suggest that DRR is proficient in reducing the impact of radiograph quality, thereby enhancing its potential in practical applications. The advantages demonstrated by DRR require further research to verify and simplify RCT and enhance the standard of diagnosis and treatment in regions with scarce medical resources.

## Data Availability

The datasets presented in this study can be found in online repositories. The names of the repository/repositories and accession number(s) can be found at: Science Data Bank (ScienceDB) CSTR: https://cstr.cn/31253.11.sciencedb.11862; DOI: https://doi.org/10.57760/sciencedb.11862.
